# New advances in drug development for metabolic dysfunction-associated diseases and alcohol-associated liver disease

**DOI:** 10.1186/s13578-024-01267-9

**Published:** 2024-07-06

**Authors:** Jinming Zhang, Yixin Li, Liu Yang, Ningning Ma, Shengying Qian, Yingfen Chen, Yajun Duan, Xiaogang Xiang, Yong He

**Affiliations:** 1 Department of Infectious Diseases, Ruijin Hospital, Shanghai Jiao Tong University School of Medicine, Shanghai, 200025 China; 2 Department of Cardiology, The First Affiliated Hospital of USTC, Division of Life Sciences and Medicine, University of Science and Technology of China (USTC), Hefei, 230001 Anhui China; 3 Shanghai Institute of Materia Medica (SIMM), Chinese Academy of Sciences, Shanghai, 201203 China; 4 University of Chinese Academy of Sciences, Beijing, China

**Keywords:** MASLD, ALD, T2DM, Metabolic disorder, Drug candidate

## Abstract

Metabolic disorders are currently threatening public health worldwide. Discovering new targets and developing promising drugs will reduce the global metabolic-related disease burden. Metabolic disorders primarily consist of lipid and glucose metabolic disorders. Specifically, metabolic dysfunction-associated steatosis liver disease (MASLD) and alcohol-associated liver disease (ALD) are two representative lipid metabolism disorders, while diabetes mellitus is a typical glucose metabolism disorder. In this review, we aimed to summarize the new drug candidates with promising efficacy identified in clinical trials for these diseases. These drug candidates may provide alternatives for patients with metabolic disorders and advance the progress of drug discovery for the large disease burden.

## Introduction

Alcohol-associated liver disease (ALD) and nonalcoholic fatty liver disease (NAFLD) share some similarities in terms of hepatic morphology and pathogenesis, and the progression of both diseases includes lipid metabolic disorders. Lipid accumulation in hepatocytes caused by chronic alcohol consumption is defined as ALD [1]. However, triglyceride accumulation in the liver and insulin resistance without other detected causes of steatosis are known as NAFLD [2]. To describe the disease more accurately and specifically, NAFLD has now been renamed as metabolic dysfunction-associated steatosis liver disease (MASLD) to define patients who have hepatic steatosis with at least one of five cardiometabolic risk factors [3]. Cardiovascular disease (CVD), type 2 diabetes mellitus (T2DM), chronic kidney disease are common extrahepatic complications in MASLD patients [4]. One-fourth of the adult population is now suffering from MASLD worldwide [5, 6]. MASLD has become the most common cause of hepatocellular carcinoma, even threatening individuals without cirrhosis [7, 8].

Similarly, Patients with ALD contributes to approximately 25% of deaths of patients with cirrhosis [9]. Considering the large burden of patients with MASLD and ALD as well as the high risk of these patients progressing to hepatocellular carcinoma (HCC), there is an urgent need to establish therapeutic strategies for these two diseases.

Diabetes mellitus (DM), a disease of the endocrine system, characterized by abnormally high blood glucose levels, is one of the most common and fastest growing diseases worldwide. It is estimated that DM will affect 693 million adults by 2045 with an increase of approximately 50% since 2017, and its prevalence is increasing annually [10]. DM with no proper treatments is life-threatening due to the persistent high blood glucose levels, which can lead to systemic vascular injury and impair the functions of the heart, eyes, kidneys, and nerves [11]. Type 2 diabetes (T2DM) is the progressive loss of insulin secretion from β-cells in the context of insulin resistance, which is commonly complicated with MASLD [12]. As the liver plays a key role in systemic metabolism, liver dysfunction is likely to contribute to insulin resistance and T2DM [13]. Clinically, patients with MASLD tend to have metabolic syndrome, such as obesity, T2DM, hyperlipidemia and hypertension [14–16]. Therefore, the demand for drugs to prevent and treat diabetes is becoming increasingly urgent.

In this review, we summarize the current drug candidates for treating MASLD and ALD to highlight promising treatments for the increasing number of patients with lipid metabolism disorders. We mainly focused on drug candidates that have been approved for phase 2 trials with good outcome to support admission to phase 3 clinical trials. In addition, we also summarize potential new targeted drugs for the treatment of diabetes. Here, we mainly focused on drugs that have been used as first-line therapy and have entered phase 2 or 3 clinical trials for the treatment of diabetes.

## Pathophysiology of MASLD and drug candidates

**Fig. 1:**
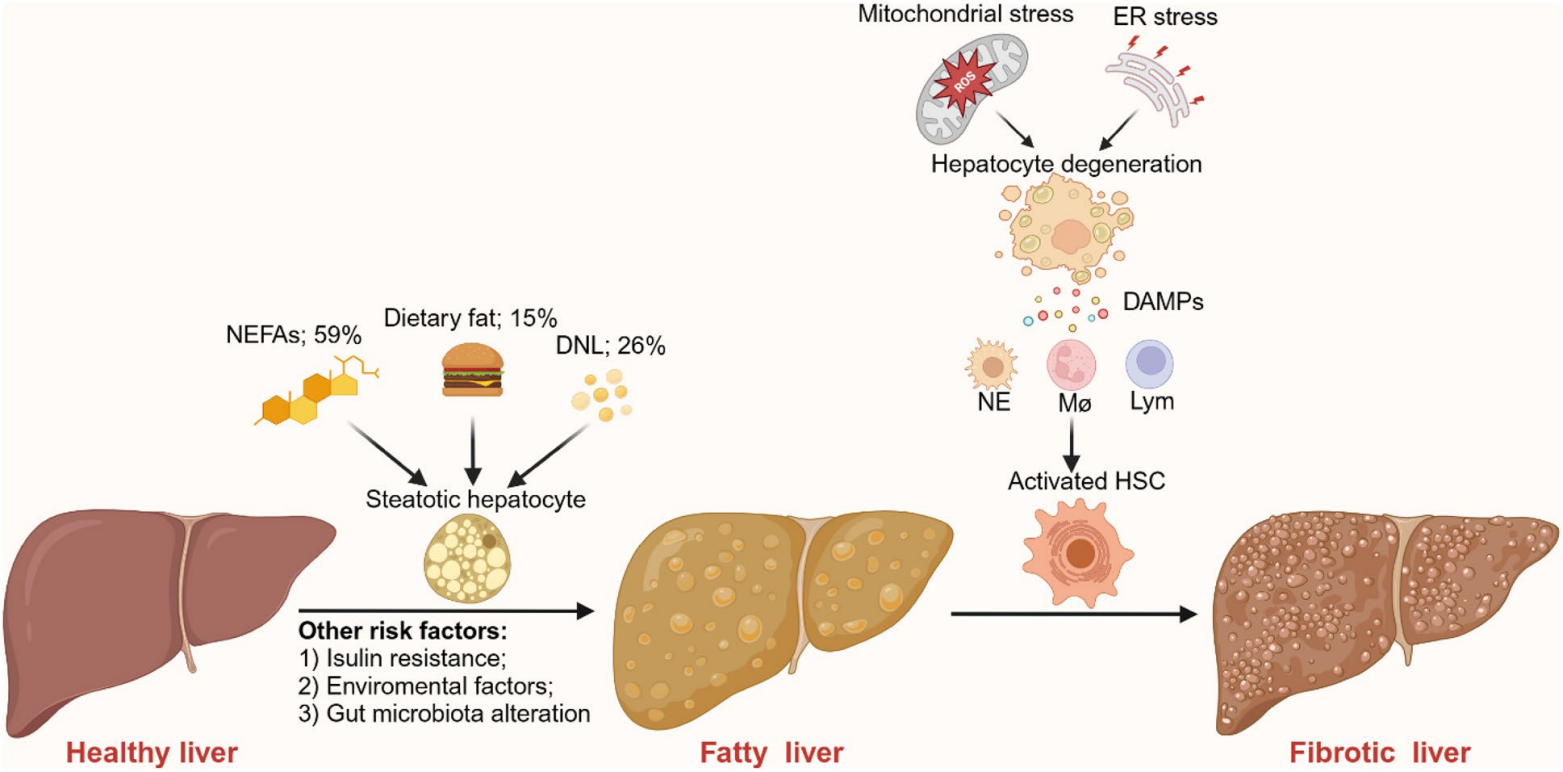
The pathophysiology of MASLD. The main pathological mechanism of metabolic dysfunction-associated steatohepatitis (MASH) involves metabolic factors and inflammatory factors. Genetic susceptibility variants, environmental factors, insulin resistance, and changes in the gut microbiota can alter lipid metabolism and induce lipid accumulation in patients with metabolic dysfunction-associated steatosis liver disease (MASLD). The primary source of intrahepatic triglyceride accumulation is hepatic uptake of plasmatic nonesterified fatty acids (NEFAs; 59%), dietary fat (15%) and de novo lipogenesis (DNL; 26%). The inflammatory response is critically responsible for the progression of MASH. Endoplasmic reticulum stress, oxidative stress and mitochondrial dysfunction lead to inflammation and hepatocyte degeneration. Injured hepatocytes release many damage-associated molecular patterns (DAMPs), recruiting various innate and adaptive immune cells to aid in damage repair, which promotes the activation of hepatic stellate cells (HSCs). HSCs migrate to injured regions and cause collagen deposition, ultimately leading to liver fibrosis

MASLD comprises a disease continuum, including steatosis with or without mild inflammation, and a necroinflammatory subtype called metabolic dysfunction-associated steatohepatitis (MASH). The main pathological mechanism of MASH comprises metabolic factors and inflammatory factors (Fig. 1). Factors such as genetic susceptibility variants, environmental factors, insulin resistance, and changes in the gut microbiota can also alter lipid metabolism and induce lipid accumulation in patients with MASLD [17]. The primary source of intrahepatic triglyceride accumulation is hepatic uptake of plasmatic nonesterified fatty acids (NEFAs; 59%), dietary fat (15%) and de novo lipogenesis (DNL; 26%) [18]. Increased levels of adipose tissue lipolysis in patients with insulin resistance also result in the upregulation of peripheral NEFA levels, which can be absorbed by hepatocytes in a concentration-dependent manner [19]. Patients with insulin resistance or type 2 diabetes mellitus (T2DM) tend to have hyperinsulinemia and hyperglycemia, promoting the hepatic conversion of carbohydrates into fatty acids by DNL [20]. Therefore, improving insulin resistance and reducing liver fat accumulation are promising targets for MASLD.

The inflammatory response is critically responsible for the progression of MASH. Mitochondrial dysfunction is reported to be involved in the advanced stage of MASH syndrome [21]. Toxic lipids in the liver, such as NEFAs, may also contribute to endoplasmic reticulum stress, oxidative stress and mitochondrial dysfunction, leading to inflammation and hepatocyte degeneration [22]. Injured hepatocytes release many damage-associated molecular patterns (DAMPs), which recruit various innate and adaptive immune cells to aid in damage repair [23]. The coexistence of chronic liver injury and repair in the progression of MASH promotes hepatic stellate cells activation and differentiation into myofibroblasts, which migrate to injured regions and cause collagen deposition, ultimately leading to liver fibrosis [24]. Advanced fibrosis is called cirrhosis and is commonly accompanied by liver decompensation and liver failure. The only efficient treatment in this stage is liver transplantation [25]. Therefore, controlling or even reversing the progression of fibrosis is essential for patients with MASH. As steatosis and fibrosis are the leading pathological alterations, the current promising therapeutic drug agents admitted into phase 3 clinical trials must contribute to either the resolution of steatohepatitis without worsening fibrosis or the reduction of fibrosis with no worsening of steatohepatitis [26].

**Fig. 2:**
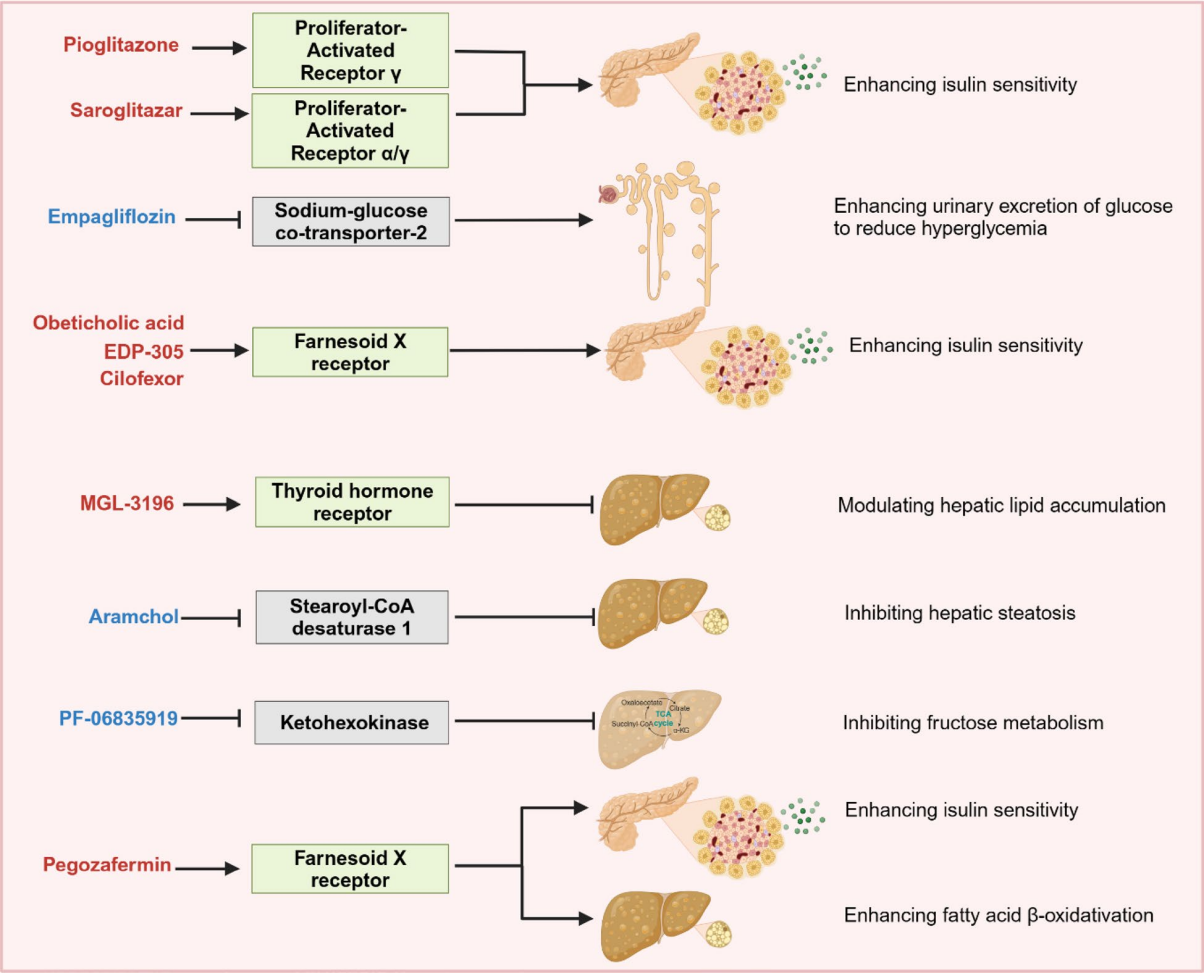
Drug candidates for MASLD. Pioglitazone and saroglitazar are promising drugs for treating MASLD because they activate PPAR receptors to enhance isulin sensitivity. Empagliflozin inhibits sodium-glucose cotransporter-2 (SGLT-2), which enhances the urinary excretion of glucose to reduce hyperglycemia. Obeticholic acid, EDP-305 and cilofor activate farnesoid X receptor (FXR) to enhance isulin sensitivity. MGL-3196 reduces hepatic lipid accumulation by activating the thyroid hormone receptor (THR). Aramchol inhibits stearoyl-CoA desaturase 1 to alleviate hepatic steatosis. PF-06835919 can inhibit ketohexokinase in the TCA cycle, subsequently controlling fructose metabolism. Pegozafermin enhances insulin sensitivity and fatty acid β-oxidation by activating farnesoid X receptor (FXR) to alleviate MASLD

There are almost no licensed treatments for MASLD so far. Adjustments in lifestyle and dietary habits, such as increasing physical activity and reducing saturated fat intake, are encouraged to curb or reverse this pathological process [13]. Lifestyle interventions contribute substantially to weight loss and improve insulin sensitivity in patients with MASLD [13]. Guidance approved by the American Association for the Study of Liver Diseases (AASLD) has stated that pharmacological treatments aimed primarily at improving liver diseases should be generally limited to those with biopsy‐proven MASH and fibrosis [27]. To date, only vitamin E, pioglitazone and Resmetirom, are recommended for pharmacological treatment in MASH patients [27, 28]. An increasing number of new drugs have entered clinical trials and shown promising therapeutic effects for MASH, which are summarized below (Fig. 2) (Table 1).

**Table 1:** Drug candidates for MASH

Drug target	Drug name	Mechanism of action	Side effect	Trial number
PPAR agonist	Pioglitazone	Enhances insulin sensitivity by activating PPARγ	Weight gain and bone loss	NCT00994682
	Saroglitazar	Improves insulin resistance and modulating gluconeogenesis, β-oxidation by activating PPARα/γ	Almost no adverse drug reactions	NCT03061721
SGLT-2 inhibitor	Empagliflozin	Downregulates renal glucose threshold and enhances urinary excretion of glucose to reduce hyperglycemia by inhibiting SGLT2	Ketoacidosis	NCT02686476
	Obeticholic acid			NCT02548351
FXR agonist	EDP-305	Improves insulin sensitivity and reduces the expression of markers of liver fibrosis and inflammation by activating FXR	High risk of long-term cardiovascular diseases	NCT03421431
THRβ agonist	Resmetirom	Modulates hepatic lipid metabolism by acting on THR; Restores RGS5 expression and inhibits STAT3 and NF-κB signaling pathway	Transient mild diarrhea and nausea	NCT03900429
SCD1 inhibitor	Aramchol	Controls hepatic steatosis by inhibiting SCD1, a central regulator of fuel metabolism	No serious adverse drug reactions	NCT02279524
Ketohexokinase inhibitor	PF-06835919	Inhibits ketohexokinase, the critical enzyme involved in fructose metabolism	No serious adverse events	NCT03256526
Fibroblast growth factor 19	NGM282	Increase metabolic rate and reduce adiposity	Increase the risk of cardiovascular disease	NCT02443116
Fibroblast growth factor 21	Pegozafermin	Regulates energy balance, glucose, and lipid homeostasis by acting on FGF21 receptor	Diarrhoea and nausea	NCT04929483

### Targeting lipometabolic disturbance and inflammation

#### PPAR agonists (pioglitazone and Saroglitaza)

PPARs are nuclear receptors. By binding fatty acids and their derivatives, PPARs are beneficial regulators of metabolic and inflammatory signaling pathways, making them promising therapeutic targets for MASLD [29]. There are three PPAR isoforms, namely, PPARα, PPARγ and PPARδ (β). PPARα mainly functions to drive genes modulating gluconeogenesis, β-oxidation, ketogenesis and lipid transport in the liver [30]. PPARγ is primarily expressed in adipose tissue and regulates glucose metabolism, lipogenesis and adipocyte differentiation [31]. In addition, PPARγ also acts as an insulin sensitizer and can prevent ectopic fat deposition [31]. Synthetic PPARα agonists and PPARγ agonists comprise the fibrates and glitazones respectively [32]. Fibrates are the first-line plasma lipid-lowering drugs for the potent ability of reducing dyslipidemia by increasing cellular fatty acid uptake, esterification and trafficking, and regulating lipoprotein metabolism genes [32]. Thiazolidinediones (TZDs) or Glitazones are clinically used for the treatment of T2DM. Mechanistically, PPARγ agonists can promote fatty acid-binding protein 4 (FABP4)-mediated free fatty acid (FFA) uptake and induce fatty acid synthase (FAS) expression to increase triglyceride in hepatocytes. PPARγ increases the transcription of sterol regulatory element-binding protein-1c (SREBP-1c), which activates other lipogenic genes and causes the conversion of pyruvate to fatty acids [33]. Weight gain as a main adverse effect restricts the use of TZDs, which has been attributed to PPARγ activation in adipose tissue. Studies have showed a critical role of PPARγ in whole-energy homeostasis [34, 35]. Two independent reports have revealed that activation of PPARγ in brain, rather than in adipose tissue, leads to TZD-induced weight gain [36, 37]. Fluid retention is a substantial side effect of TZDs, which would increase the risk for adverse cardiovascular events, especially congestive heart failure. The mechanism by which TZDs leads to this consequence may attribute to the altered sodium and water reabsorption in the distal collecting ducts of kidney [38, 39]. High rate of fractures in human patients is another serious side effect of TZDs [40]. Moreover, studies have showed that bone cell-autonomous PPARγ action results in TZD-induced bone disease, which promotes osteoclastogenesis while inhibiting osteoblastogenesis [41–43]. Hepatotoxicity has beseted the development and application of TZDs in clinical. Troglitazone as one of TZDs, previously used for the treatment of non-insulin-dependent diabetes mellitus has been withdrawn from the market in 2000 for the severe drug induced-liver failure [44]. The roles of PPARδ remain to be further explored, but it has been reported that activation of PPARδ promotes hepatic fatty acid oxidation and limits inflammation [31].

Pioglitazone is an endorsed TZDs for MASH, an activator of PPARγ. By enhancing insulin sensitivity, activation of PPARγ can reduce plasma levels of FFAs and decrease hepatic lipid accretion [45]. As a kind of thiazolidinedione, pioglitazone has been demonstrated to be beneficial for improving histopathological components of MASH, including reducing inflammation and hepatocyte degeneration, while its consistent efficacy in limiting fibrosis has decreased [46]. The application of thiazolidinedione directly inhibits the activation of HSCs and the progression of fibrosis in rats [47]. A meta-analysis showed that pioglitazone significantly improved ballooning (RR 1.62 and OR 2.11), steatosis (RR 2.03 and OR 3.39), inflammation (RR 1.71 and OR 2.58) and fibrosis (RR 1.38 and OR 1.68) in MASH patients [48]. Genetic analysis has identified a variant of CYP2C8, encoding the predominant pioglitazone-metabolizing enzyme, which is associated with fibrosis score [49]. Weight gain, bone loss, fluid retention and heart problems are the mian side effects limiting the widespread use of pioglitazone [50]. As TZDs acts on a variety of tissues to confer insulin sensitization, causing a series of deleterious side effects, developing tissue-specific compounds is urgent need.

The dual PPARα/γ agonist Saroglitazar has been authorized for the treatment of MASH by the Drug-Controller General of India (DCGI) [51]. Preclinical data have revealed that saroglitazar improved steatosis, lobular inflammation, hepatocellular ballooning and fibrosis in diet-induced mouse models of MASLD [52]. However, Saroglitazar is still in the phase 2 stage in America and has not been approved by the Food and Drug Administration (FDA). A phase 2 clinical trial has indicated that 16 weeks of 4 mg Saroglitazar treatment markedly improves alanine aminotransferase (ALT) levels and insulin resistance in a cohort of 106 patients with MASLD/MASH syndrome [53]. Another phase 2 double-blinded clinical trial has also confirmed that Saroglitazar could improve histological parameters and alter the lipoprotein profile in patients with MASH syndrome, and this study has also demonstrated that Saroglitazar is well tolerated when administered and that no adverse events are directly associated with it [54].

Therefore, Saroglitazar is a promising drug agent for the treatment of patients with MASH, but a large cohort of phase 3 clinical trials needs to be conducted to confirm the long-term efficacy and safety of this drug in MASH.

### Targeting glucose and lipid metabolism disorders

#### SGLT-2 inhibitors

Glucose can be completely reabsorbed in the proximal tubule in healthy people. The early proximal tubules (S1 and S2 segments) are the main place for glucose absorption and the later segment (S3) is responsible for the rest [55]. In T2DM, both liver and kidney contribute to excess glucose production, and up to ~ 20% of the total glucose from renal glucose reabsorption is released to the circulation [56]. Reabsorption of glucose filtered by the glomeruli substantially affects the circulating glucose pool, which might actually be increased in T2DM [57]. SGLT (sodium-glucose cotransporter) proteins are encoded by the solute carrier 5 (*SLC5*) subfamily of sodium/substrate symporter genes [58]. SGLT1 and SGLT2 are the most well characterized proteins encoded by *SLC5* genes, which are sodium-dependent glucose transporters for the reabsorption of glucose [58]. SGLT1 is expressed in the more distal segments (S2 and S3) of the proximal convoluted tubule, where it mediates the reabsorption of glucose that has been reabsorbed earlier in the tubule by SGLT2 [55]. SGLT-2 is almost entirely confined to the first segment (S1) epithelium of the kidney and responsible for the reabsorption of the majority (90%) of the glucose filtered by the kidneys [59]. Thus, developing SGLT-2 inhibitors to reduce hyperglycemia are promising therapeutic strateges for T2DM patients.

SGLT-2 inhibitors, such as dapagliflozin, canagliflozin, empagliflozin and ipragliflozin, downregulates the reabsorption ability while increases urinary excretion of glucose in patients with T2DM [60]. Several clinical trials have been conducted to examine the efficacy of SGLT-2 inhibitors in patients with MASLD and T2DM, and the results has indicated that SGLT-2 inhibitors (canagliflozin, dapagliflozin or empagliflozin) significantly improves hepatic lipid deposition, liver enzyme levels and liver stiffness [61–65]. In addition, the application of pioglitazone in biopsy-confirmed MASH patients with or without T2DM can significantly alleviate lobular inflammation and MASLD activity scores and contribute to MASH resolution without aggravating fibrosis [46, 66–69]. SGLT2 inhibitors contribute to weight loss, which is strongly associated with a decrease in hepatic lipid loss [61], and MASLD patients combined with T2DM have a tendency to ketoacidosis when administrated with Empagliflozin [70]. Overall, SGLT2 inhibitors show promising efficacy in patients with MASH by reducing hepatic lipid accumulation, but more histological evidence is needed to confirm the benefits of SGLT2 inhibitors in hepatic steatosis.

#### FXR agonists (OCA, EDP-305, cilofexor)

Farnesoid X receptor (FXR) is a bile acid (BA)-activated nuclear transcription factor mainly expressed in the liver, ileum, kidney, and adrenal glands [71]. FXR actions are comprehensive and complicated, as FXR regulates more than 300 response genes directly and possibly thousands of genes indirectly by binding the retinoid X receptor and a variety of FXR response elements as a monomer, homodimer or heterodimer [72–74]. Accordingly, multiple metabolic pathways are regulated by FXR response [72–74].

FXR not only controls BA synthesis, transport and enterohepatic circulation [75, 76] but also acts as a key regulator in lipid and glucose metabolism [77, 78]. In addition, FXR activation exert powerful immunomodulatory function, inhibiting innate and adaptive immune responses [79, 80]. FXR activation inhibits BA synthesis by upregulating fibroblast growth factor (FGF) 19 expression and downregulating the rate-limiting enzyme cytochrome P450 7A1 (CYP7A1) [81]. FXR plays a primary role in lipid regulation. FXR activation suppresses the expression of SREBP-1c in hepatocytes, which is a critical transcription factor that modulates lipogenesis by inducing key enzymes, such as FA synthase [77]. In human cells, FXR activation can induce expression of PPARα as well as its target genes to promote FFA oxidation [82]. FXR can also induce FGF21. Expressed in hepatocytes and acting in the central nervous system and adipose tissue, FGF21 controls glucose, lipid, and energy metabolism. FGF21 analogues have been demonstrated to decrease steatosis, inflammation, and fibrosis in NASH models [83]. FXR regulates glucose metabolism through modulating gluconeogenesis and glycogenolysis in the liver, and controlling peripheral insulin sensitivity in striated muscle and adipose tissue [78]. FXR knock out mice show elevated serum glucose and impaired glucose and insulin tolerance with decreased inhibition function of hepatic glucose production by insulin and increased peripheral glucose levels [78]. In human, FXR activation can improve insulin sensitivity and reduce serum liver inflammatory markers in patients with type 2 diabetes and NASH [84]. FXR activation can also inhibit immune responses associated with chronic inflammation in NASH. By inhibiting nuclear factor kappa-B (NF-κB) [85], a critical transcription factor in inflammation, FXR signaling impairs neutrophil accumulation and reduces pro-inflammatory mediators [79]. In addition, FXR activation has been demonstrated to inhibit the expression of monocyte chemoattractant protein 1 (MCP-1, also called as CCL-2) [86], which is an important chemokine involved in the progression of NASH [87]. Therefore, FXR signaling shows complicated functions both in metabolism and inflammation.

OCA (Obeticholic acid) is a bile acid derivative. As an endogenic agonist of FXR, OCA mediates lipid metabolic signaling pathways. The application of OCA in patients with MASLD and T2DM contributes to the improvement of insulin sensitivity and a reduction in the expression of markers of liver fibrosis and inflammation [84]. Clinical trials, including the REGENERATE study, have indicated that OCA can significantly improve liver fibrosis and the key components of MASH disease activity, suggesting that OCA clinically improves hepatic histology in patients with MASH [88, 89]. However, evidence for long-term clinical outcomes and safety needs further verification [88, 89]. Moreover, data from a new efficacy and safety analysis of the REGENERATE trial has confirmed that the antifibrotic effect of 25 mg OCA and OCA is generally well tolerated over long-term dosing [90]. Common adverse effect of FXR agonists is only mild to moderate generalized pruritus in a minority of patients [91]. In conclusion, OCA shows encouraging efficacy by inhibiting liver fibrosis in a dose-dependent manner and can be well tolerated with a favorable safety profile.

EDP-305 and cilofexor, which are FXR synthetic agonists, have also been evaluated in clinical trials in patients with MASH syndrome. EDP-305 is an oral FXR agonist under development for the treatment of MASH. The results of a double-blind phase 2 study of EDP-305 suggested that treatment with EDP-305 for 4 months can reduce ALT levels and hepatic fat content in 134 patients with fibrotic MASH syndrome [92]. Given this, a longer-term trial of EDP-305 is needed to assess histological endpoints in patients with MASH. Moreover, the nonsteroidal FXR agonist cilofexor is site-specific and contributes to reducing liver lipid content in patients with MASH without altering blood levels of lipids or disturbing insulin resistance [93].

Currently, the development of systemic FXR agonists for metabolic liver disease is challenging due to adverse effects, including increased levels of cholesterol, low-density lipoprotein cholesterol (LDL-c) and high-density lipoprotein cholesterol (HDL-c), which raises concerns regarding long-term cardiovascular safety [94]. Several FXR agonists are being combined with other agents, including cenicriviroc, a CCR2/CCR5 inhibitor [94], or firsocostat, an acetyl-CoA carboxylase inhibitor, to treat MASH [95, 96]. However, the efficacy and safety of these combination therapies need further evaluation.

#### THRβ agonist (MGL-3196)

Thyroid hormone receptors (THRs) are nuclear hormone receptors that mediate hepatic lipid metabolism [97]. THRα and THRβ are two isoforms encoded by THR genes (*THRA* and *THRB*, respectively) [98]. In particular, THRβ is primarily expressed in the liver and is closely associated with hepatic lipid metabolism [99]. Mechanistically, an animal study has revealed for the first time that THRβ-mutant mice exhibit increased activation of PPARγ signaling and inhibition of THR-mediated fatty acid β-oxidation results in hepatic lipid accumulation [100]. However, unselective THR targeting can be accompanied by negative side effects due to high THRβ expression in other organs and THRα-mediated effects [101]. Resmetirom is an oral, liver-targeted, THRβ–selective agonist in development for the treatment of NASH with liver fibrosis [102]. Mechanically, Resmetirom suppresses signal transducers and activators of transcription 3 (STAT3) and NF-κB signaling pathways in an regulator of G protein signaling 5 (RGS5)-dependent manner in mouse models [103].

Resmetirom has been evaluated in patients with MASH syndrome in a phase 2 trial, and the results suggest that Resmetirom can significantly reduce hepatic fat accumulation after 12 and 36 weeks of treatment [104]. Further studies have assessed the efficacy and safety of Resmetirom and revealed that Resmetirom is well tolerated, with only transient mild diarrhea and nausea, and further phase 3 MASH studies should be conducted [105]. Moreover, Recently, an ongoing phase 3 trial of a large enrolled population has consolidated the evidence that Resmetirom is superior to placebo in improving MASH outcome [102]. Till now, Resmetirom has become the only drug approved by FDA for the treatment of adults with noncirrhotic NASH with moderate to advanced liver fibrosis (consistent with stages F2 to F3 fibrosis) [106].

#### SCD1 inhibitor (aramchol)

Stearoyl-CoA desaturase 1 (SCD1) as a rate-limiting enzyme, which is highly expressed in lipogenic tissues, including adipose tissue and liver, mainly modulates the biosynthesis of monounsaturated fatty acids (MUFAs) [107]. MUFAs not only have structural functions, but also are involved in regulating systemic metabolism and modulating chronic metabolic diseases [107].

The different expression locations of SCD1 may show various functions. Global knock out of SCD1 can be protected from high carbohydrate diet (HCD) and high fat diet (HFD)-induced adiposity and hepatic steatosis [108]. SCD1 global knock out mice also displays enhanced utilization of glucose in skeletal muscle and heart with increased insulin signaling in these tissues [109, 110], which is mainly attributed to the hypermetabolic condition observed in the mice. While hepatic SCD1 specific-deficient mice can only be protected from HCD but not HFD induced adiposity and hepatic steatosis [111]. Hepatic SCD1 deficiency mice exhibit a significant reduction in hepatic lipogenic gene expression and a reduced de novo lipogenesis associated with reduced hepatic triglyceride (TG) secretion. However, combined deletion of SCD1 from liver and white adipose tissue (WAT) fails to protect mice from HFD induced adiposity [112]. Interestingly, SCD1 skin-specific knock out mice shows a semblable phenotype with global SCD1 deficiency, which shows a hyperphagic and maintained lean phenotype accompanied with protection against extended HFD feeding-induced insulin resistance [113]. In summary, as a central regulator of fuel metabolism, SCD1 may be a therapeutic target for controlling hepatic steatosis.

Patents with MASH tend to have increased de novo lipogenesis. Elevated levels of SCD1 activity have been detected in patients with MASLD [114]. Aramchol, an SCD1 inhibitor, has been administered to patients with MASH syndrome. In addition to reducing hepatic fat accumulation, Aramchol can directly attenuate cellular fibrogenesis by downregulating the expression level of *SCD1* mRNA and elevating *PPARG* (PPAR gamma) in HSCs, contributing to a reduction in the expression of *COL1A1* (Collagen type I alpha 1) and *ACTA2* (Actin Alpha 2, Smooth Muscle) [115]. Importantly, data from a phase 2 trial has indicated that Aramchol 600 and 400 mg are safe, well tolerated in patients with MASH and can improve liver histology and enzymes [116]. However, a reduction in liver fat, the primary endpoint, was not significantly different between the Aramchol treatment group and the placebo-control group [116]. A phase 3 study evaluating the long-term efficacy and safety of aramchol in patients with MASH syndrome is currently ongoing [116]. Therefore, aramchol may be a therapeutic agent for the treatment of MASH, but more clinical and histological evidence should be collected in future studies.

#### Ketohexokinase inhibitor (PF-06835919)

In the liver, fructose can serve as a substrate for de novo lipogenesis, resulting in intrahepatic lipid accumulation. Ketohexokinase (KHK) catalyzes the phosphorylation of fructose to form fructose-1-phosphate, a critical enzyme involved in fructose metabolism [117]. Excessive fructose consumption induces an increase in KHK expression, hepatic steatosis, and impaired fatty acid oxidation, contributing to the development of MASLD [118]. The inhibition of KHK has been demonstrated to improve steatosis, fibrosis, and inflammation in preclinical studies [119].

At present, PF-06835919, a KHK inhibitor, has entered clinical trials [120]. The results of a phase 2 trial showed that a KHK inhibitor may benefit adults with MASLD and insulin resistance [120]. Recently, a phase 2a clinical trial has indicated that the administration of PF-06835919 for 16 weeks is generally safe and well tolerated, and PF-06835919 treatment also reduces whole liver fat, as determined by MRI of the proton density fat fraction (PDFF) [121]. Overall, KHK inhibitors may have therapeutic effects on patients with MASLD and T2DM. Till now, no serious adverse events of PF-06835919 have been reported. However, the clinical benefits of PF-06835919, including the outcome and safety of its long-term use, need further investigation. Moreover, there is no clinical evidence that KHK inhibitors can alleviate inflammation in these patients.

#### Fibroblast growth factor 19 (FGF19)

Human fibroblast growth factor (FGF) 19 and its mouse ortholog FGF15 belong to a subfamily of FGFs. FGF15/19 is induced by FXR activation after the postprandial reuptake of bile acids and expressed in ileal enterocytes of the small intestine [122]. The secreted FGF15/19 represses bile acid synthesis and gluconeogenesis and promotes glycogen and protein synthesis [123]. The elevated BA level induces the production of FGF15/19 and then FGF15/19 inhibits the expression of rate-limiting enzyme CYP7A1 of BA synthesis [122]. Pharmacologically use of FGF15/19 can also act on central nervous system, brown adipose tissue (BAT) and WAT. Chronic exposure to FGF19 is able to increase metabolic rate and reduce adiposity [124, 125]. Signaling by the endocrine FGF15/19 requires not only its FGF receptors (FGFRs) but also a coreceptor, a single-transmembrane protein from Klotho family. FGF15/19 binds to βKlotho in complex with FGFR1c, 2c, 3c, and 4 [126], and then activates extracellular signal-regulated kinases 1 and 2 (ERK1/2) and other downstream kinases. Importantly, FGF15/19 can act on hepatocytes directly to inhibit CYP7A1 by activating ERK 1/2 [127]. In addition to its beneficial effects on liver metabolism, FGF15/19 also stimulates hepatocyte proliferation through an FGFR4-dependent mechanism, resulting in hepatocellular carcinomas [128, 129].

The administration of NGM282, an FGF-19 analog, over 12 weeks in patients with MASH syndrome has been demonstrated to be generally tolerated but has not resulted in a significant dose‒response effect on improving fibrosis in at least one stage [130]. However, administration of NGM282 inhibits cholesterol metabolism, leading to an elevated level of LDL-C [131]. This adverse effect may increase the risk of cardiovascular disease (CVD) [131]. Although it has been reported that pharmacologically use of FGF15/19 leads to liver cell growth and neoplasia [128], NGM282, a full FGFR4 agonist, due to its inability to activate STAT3 pathway, it lacks tumor-promoting activity in mice [126, 132].

#### Fibroblast growth factor 21 (FGF21)

Unlike FGF15/19, FGF21 is expressed in various tissues, such as liver, BAT, WAT and pancreas [133]. FGF21 is shown to be significantly elevated upon food deprivation and feeding ketogenic diet in rodents [134]. FGF21 also binds to βKlotho in complex with FGFR1c, 2c, or 3c, but not 4 [126]. Metabolism-related functions of FGF21 includes inducing fatty acid oxidation, ketogenesis, and gluconeogenesis as well as suppressing lipogenesis [134–138]. In contrast to FGF15/19, the effects of FGF21 on the liver appears to be indirect and the in vivo effects of FGF21 have not been recapitulated in either isolated, perfused livers or primary rodent hepatocytes treated with FGF21 [136, 139]. FGF21 can induce the expression of uncoupling protein 1 (*Ucp1*), which is typically found in brown adipocytes, driving the so-called “browning” of white adipocytes [140]. This likely contributes to FGF21 thermogenic effects [140]. Adiponectin, a hormone that regulates glucose and fatty acid homeostasis, is also induced by FGF21 in white adipocytes and is required for the full metabolic efficacy of FGF21 in vivo [141, 142]. Mechanistically, FGF21 enhances the phosphorylation of the transcription factor cAMP response element-binding protein (CREB), which directly modulates *Ucp1* expression [143]. FGF21 also increased the phosphorylation of STAT3, leading to mitochondrial respiration [143]. These findings underline the significant role of FGF21 in enhancing thermogenesis by acting directly on BAT.

FGF21 plays an important role in regulating energy balance, glucose and lipid homeostasis, exerting beneficial effects on metabolism in individuals with obesity and T2DM [144]. Importantly, the administration of FGF21 does not lead to adverse effects of hypoglycemia or mitogenesis in rodents or monkeys [144]. Phase 2 trials have demonstrated that the FGF21 analogs pegozafermin and efruxifermin significantly improve liver fibrosis and reduce the hepatic fat fraction (HFF) and are generally well tolerated, supporting the phase 3 trial of this drug in a large cohort [145–147]. A recent randomized, controlled trial has reported that the most common adverse events of pegozafermin are nausea and diarrhea [148]. Although the encouraging pharmaceutical efficacy of FGF21 analogs has been shown in several clinical trials, the long-term impacts on histopathology, outcome, and safety in patients with MASH syndrome have not been illustrated.

## Pathophysiology and drug candidates for ALD

**Fig. 3:**
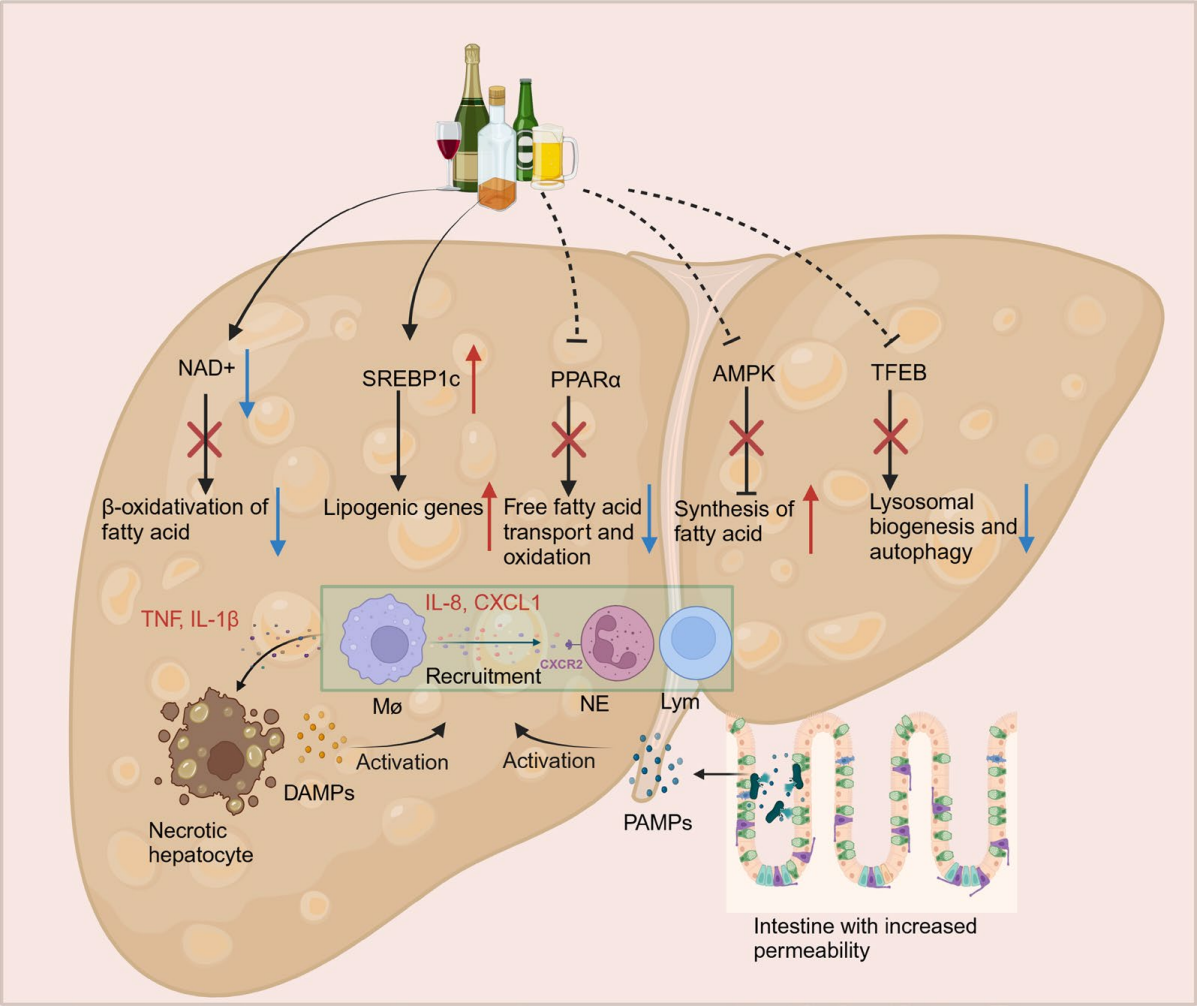
The pathophysiology of ALD. The mechanisms of hepatic fat accumulation caused by alcohol consumption are mainly attributed to the following four factors: (1) Alcohol oxidation increases the NADH: NAD^+^ ratio, thus promoting alcohol oxidation at the expense of fatty acid oxidation. (2) Hepatic triglyceride accumulation mainly attributes to the action of SREBP1c and ethanol can induce the upregulation of SREBP1c. (3) Ethanol feeding impairs the activation of PPARα, which has been demonstrated to protect against alcohol-induced liver injury by promoting free fatty acid transport and oxidation. (4) Alcohol impairs the function of AMPK, which can protect against alcohol-induced liver injury by inhibiting the synthesis of fatty acid. The consumption of alcohol strongly alters intestinal permeability and hepatocyte death. Subsequently, PAMPs, including phosphate lipopolysaccharide (LPS) derived from the gut and DAMPs released by damaged hepatocytes, stimulate innate immune cells and adaptive immune cells, contributing to increased cytokine and chemokine production and liver inflammation. Moreover, Ethanol feeding decreases hepatic expression of TFEB, which is required in lysosomal biogenesis and autophagy

ALD comprises several major stages, including simple fatty liver disease, alcoholic steatohepatitis (ASH), liver fibrosis, cirrhosis, and hepatocellular carcinoma [149, 150]. Chronic ASH ultimately leads to fibrosis and cirrhosis, while severe ASH leads to acute alcoholic hepatitis (AH), which has a poor prognosis. AH occurs in the presence of cirrhosis and is defined as an acute-on-chronic disease with a more severe inflammatory response and poorer prognosis. Lipid metabolic alternations are the early pathophysiological response to chronic alcohol consumption in ALD, resulting in hepatic steatosis. The mechanisms of hepatic fat accumulation caused by alcohol consumption are mainly attributed to the following four factors (Fig. 3): (1) alcohol consumption leads to an elevated ratio of NADH/NAD^+^ in hepatocytes, interrupting mitochondrial β-oxidation of fatty acids [151]; (2) alcohol consumption upregulates the expression of SREBP1c in the liver, which serves as a key transcription factor that promotes lipogenic gene expression [152]; (3) PPARα, which upregulates the expression of genes involved in free fatty acid transport and oxidation and can be inactivated by alcohol [153]; (4) alcohol impairs the function of 5ʹ-AMP-activated protein kinase (AMPK), which ultimately leads to the upregulation of downstream synthesis of fatty acid [154]. The consumption of alcohol strongly leads to the alteration of intestinal permeability and hepatocyte death. Subsequently, PAMPs, including phosphate lipopolysaccharide (LPS) derived from the gut and DAMPs released by damaged hepatocytes, activate innate immune cells and adaptive immune cells, contributing to increased cytokines and chemokines, such as tumour necrosis factor (TNF), Interleukin (IL)-1β, chemokine (C-X-C motif) ligand 1 (CXCL1) [155]. TNF and Interleukin-1 beta (IL-1β) induce hepatocytes necrosis. IL-8 and CXCL1 released from macrophages recruit neutrophils by CXC chemokine receptor 2 (CXCR2). Moreover, Ethanol feeding decreases hepatic expression of transcription factor EB (TFEB), which is required in lysosomal biogenesis and autophagy [156].

Strategies for the treatment of ALD differ according to the clinical course. Patients with compensated ALD are treated with motivational therapy and considered drugs for achieving alcohol abstinence. When progressing to decompensated ALD with superimposed AH, existing hepatocytes cannot support all the functions of the liver, and patients in this stage often have obvious and severe clinical manifestations with high mortality. In fact, liver transplantation is the only choice for highly selected patients for whom no other therapeutic strategies are available [157]. As there are currently few treatments for AH and corticosteroids are the only drugs approved for severe AH by AASLD with controversial efficacy on AH, we have summarized the potential and promising drug candidates here.

### Targeting lipid metabolism and inflammation

#### Corticosteroids

Corticosteroids are powerful anti-inflammatory drugs. As several inflammatory mediators are induced during the progression of AH, the administration of corticosteroids in AH is considered an appropriate therapeutic strategy for treating this disease. However, the results of a series of clinical trials have shown that the efficacy of corticosteroids in AH is controversial. Several meta-analyses have demonstrated that the application of corticosteroids in patients with AH can prolong survival time [158, 159], but collective data do not support their wide use due to the heterogeneous implementation of clinical trials and bias. Another meta-analysis of individual patient data from 11 randomized controlled trials showed that the efficacy of corticosteroids in AH patients is limited and that corticosteroid use only reduces the risk of death within 28 days of treatment but not within the following 6 months [158]. In addition, the administration of corticosteroids should be intensively monitored for infection because of their extensive anti-inflammatory effects, as corticosteroids are not recommended for patients with severe infection. Therefore, the limited use of corticosteroids with controversial efficacy in patients with AH calls for more new drugs.

#### Larsucosterol

Larsucosterol is an endogenous cholesterol derivative of 25-hydroxycholesterol 3-sulfate (25HC3S). 25HC3S can reduce intracellular lipid accumulation by inhibiting lipogenesis. Moreover, 25HC3S alleviates inflammation by suppressing inflammatory mediators and plays a role in inhibiting apoptosis [160]. Research has also shown that 25HC3S protects against DNA damage by reducing hypermethylation [161]. A pilot study has demonstrated that Larsucosterol shows promising efficacy in patients with AH without safety concerns. Larsucosterol is now in a phase 2b clinical trial with a larger cohort to test its efficacy and safety [162].

#### Anakinra

IL-1β is a potent proinflammatory cytokine that is upregulated in patients with ALD. A mechanistic study shows that IL-1β activates the inflammasome in bone marrow-derived Kupffer cells [163]. Blocking IL-1β signaling markedly mitigates inflammation, steatosis, and fibrosis in a mouse model of ALD [163], which indicates that IL-1β is a potential target for AH. A multicenter randomized clinical trial showed that combination treatment with an IL-1 receptor antagonist (anakinra) and pentoxifylline plus zinc can improve survival in patients with alcohol-associated hepatitis in a manner similar to the benefits of corticosteroid therapy [164], although pentoxifylline did not improve survival in patients with alcoholic hepatitis [165].

### Regenerative agents

#### Interleukin-22 (IL-22)

IL-22 is a member of the interleukin-10 (IL-10) family that is secreted by activated T cells, including T helper 22 (Th22) cells, Th17 cells and Th1 cells, as well as subsets of innate lymphoid cells. However, IL-22 only targets certain nonhematopoietic tissues of the respiratory and gastrointestinal systems [166]. IL-22 protects against hepatocyte damage and promotes cell proliferation [167, 168]. Moreover, IL-22 has been demonstrated to induce the production of antibacterial proteins in hepatocytes in mice with acute-on-chronic liver failure [169]. Treatment with IL-22 in a murine model of chronic-plus-binge ethanol feeding has shown that IL-22 activates hepatic STAT3 and improves alcoholic fatty liver, liver injury, and hepatic oxidative stress [170].

A phase-2 dose-escalation study indicated that the administration of 45 μg/kg IL-22Fc (F-652), a recombinant fusion protein consisting of two human IL-22 molecules linked to an immunoglobulin constant region (IgG2-Fc) with an extended half-life, can significantly improve MELD scores and promote hepatic regeneration as well as reduce inflammatory markers [171], supporting the further study of randomized placebo-controlled trials to confirm the efficacy of F-652 in AH. Therefore, IL-22 has the potential to be a promising therapeutic candidate for AH when used alone or combined with prednisolone.

#### G-CSF

Granulocyte colony-stimulating factor (G-CSF) is a glycoprotein that induces the production of neutrophils and stem cells (CD34^+^) in the bone marrow and promotes the migration of these cells to peripheral blood [172]. The administration of G-CSF has been demonstrated to mobilize pluripotent cells, which help induce liver regeneration and improve the survival rate [173].

Clinical trials of small cohorts have shown that the administration of G-CSF to patients with severe alcoholic hepatitis is safe, reduces disease severity and 90-day mortality and promotes the proliferation of hepatocytes, suggesting the safety and therapeutic efficacy of G-CSF in these patients [174–176]. A meta-analysis has revealed that the administration of G-CSF is associated with a mortality reduction of more than 70% in 3 months among patients with AH [172]. However, due to the lack of preclinical evidence that G-CSF is effective in mouse models of alcoholic hepatitis, clinical trials with large cohorts are needed to test its efficacy in patients with AH.

## Pathophysiology and therapeutic drugs for T2DM

**Fig. 4:**
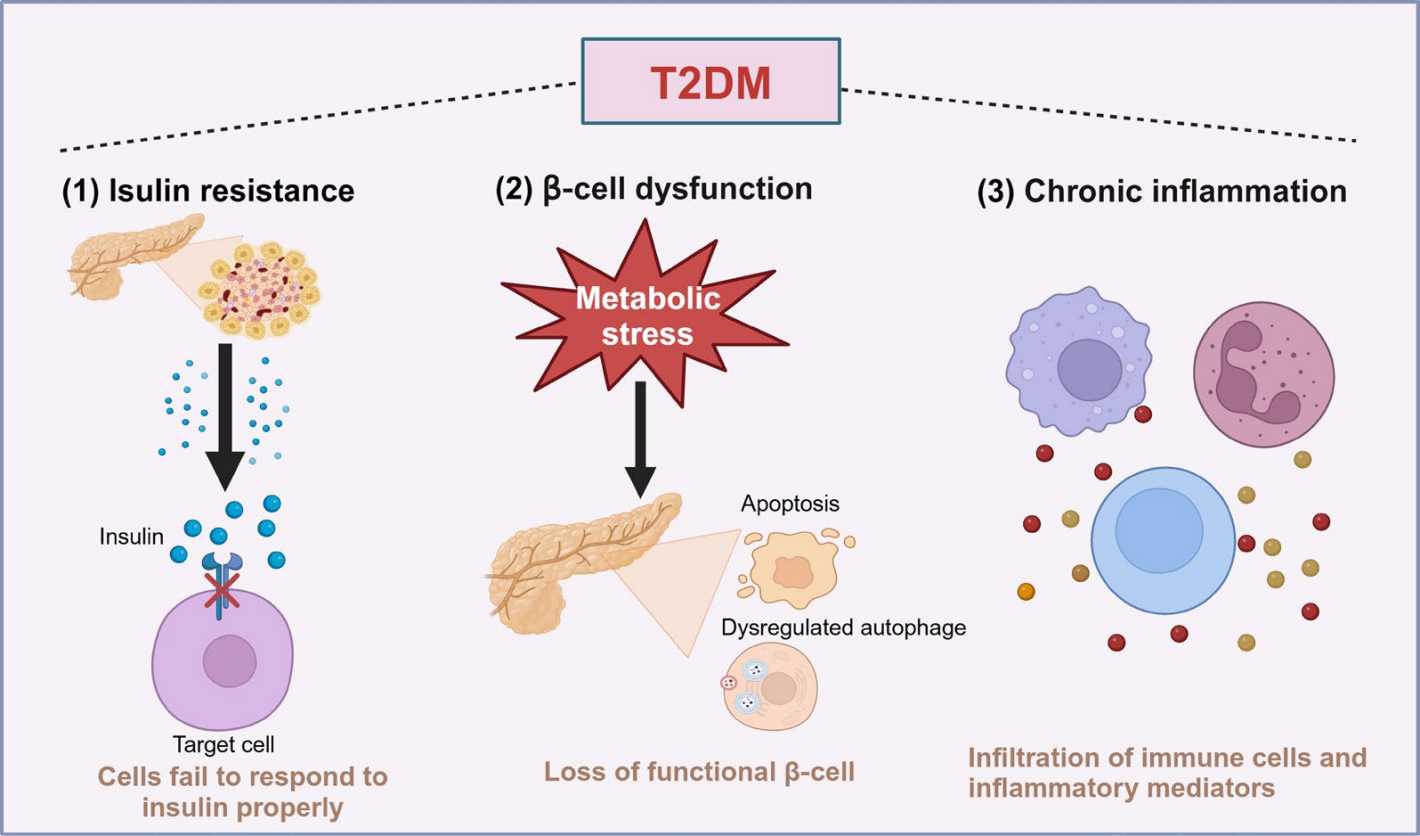
Pathophysiological mechanisms of T2DM. Insulin resistance, β-cell dysfunction and chronic inflammation are the three main pathophysiological changes in T2DM. (1) In T2DM, although insulin is released by pancreas islet β cell, the target cells can not respond to insulin; (2) Metabolic stress leads to the apoptosis and dysregulated autophagy of β cells, contributing to the reduced functional β cells; (3) The increased infiltration of immune cells, such as macrophages and T cells, results in the increased levels of inflammatory mediators

T2DM is characterized by dysregulation of carbohydrate, lipid and protein metabolism with impaired control of hyperglycemia [177]. Insulin resistance, β-cell dysfunction and chronic inflammation are the three main pathophysiological changes in T2DM, which hamper the control of blood glucose levels and result in the progression of micro- and macrovascular complications (Fig. 4). T2DM accounts for more than 90% of all cases. The pathogenesis of insulin resistance is complicated and multifactorial. As T2DM clusters in families and is heritable, genetic abnormalities are the primary pathogenesis of insulin resistance, ultimately resulting in Islet B cell failure [178]. Obesity and physical inactivity also act as co-factors lead to insulin resistance with a genetic predisposition [179].

Diabetic angiopathy is the main complication of T2DM, which is closely linked to the severity and duration of hyperglycemias [10, 180]. Retinopathy and nephropathy are the typical peripheral microvascular diseases in patients with T2DM. Accelerated atherosclerotic cardiovascular, higher rates of hypertension, myocardial infarction, stroke, peripheral vascular disease and congestive heart failure are the life-threatening macrovascular complications in patients with T2DM. In regard of the serious consequences of this chronic disease, we have summarized the promising drug candidates for the treatment of T2DM (Table 2).

**Table 2:** Drug candidates for diabetes

Drug target	Drug name	Mechanism of action	Study population
Not clear	Metformin	By activating AMPK signaling pathway, lipogenesis can be reduced and insulin sensitivity can be improved	T2DM, MASLD
SGLT2	Canagliflozin	Inhibits SGLT2, enhance glucose excretion by reducing the reabsorption of glucose by the kidney, and thus play a hypoglycemic role	T2DM, Obesity
	Dapagliflozin Ertugliflozin		
PPAR agonist	Pioglitazone	Regulate the transcription of insulin-responsive genes, thereby regulating glucose production, transportation and utilization	T2DM, Obesity
GLP-1 receptor	Exenatide	Mainly to stimulate insulin secretion in a glucose-dependent manner	T2DM
	Liraglutide		
DDP-4	Sitagliptin	By inhibiting the activity of DPP4, the hydrolysis of DPP4 is reduced, thus lowering blood glucose	T2DM
G-protein-coupled receptors	TAK-875	Stimulates β cells to secrete insulin	T2DM

### Metformin

Metformin has been used as a hypoglycemic drug for diabetic patients for more than 50 years. It is the most commonly used first-line drug in the treatment of T2DM [181]. There are many mechanisms of metformin. It is reported that metformin can reduce fat production and improve insulin sensitivity by activating AMPK signaling pathway, and can also inhibit gluconeogenesis by inhibiting mitochondrial redox shuttle [182]. In a clinical study, 14,847 patients with T2DM and sepsis were divided into two groups. One group of 682 patients (4.6%) received metformin treatment during hospitalization, and the other 14,165 patients (95.4%) did not receive treatment. The results showed that metformin not only alleviated the symptoms of diabetes but also reduced the 90-day mortality rate as well as the severe acute kidney injury [183].

### SGLT2 inhibitors

In patients with T2DM, SGLT2 inhibitors can increase glucose elimination by inhibiting the expression of SGLT2 protein and decreasing the reabsorption of glucose by the kidney, thus reducing hyperglycemia and improving the insulin secretion and peripheral insulin sensitivity [184]. SGLT2 inhibitors (gliflozins) are a new class of oral hypoglycemic drugs, which can inhibit SGLT2 in proximal tubules, and enhance glucose excretion by reducing glucose reabsorption in the kidney [185]. Other SGLT2 inhibitors, such as Canagliflozin, Dapagliflozin, Ertugliflozin, and Sotagliflozin can also play a hypoglycemic role by inhibiting the reabsorption of glucose from urine [186, 187].

### Thiazolidinediones (TZDs) insulin sensitizers

TZDs do not directly stimulate insulin secretion, but mainly increase the sensitivity of target organs such as skeletal muscle, liver and adipose tissue to insulin. In addition, TZDs can indirectly achieves the effect of lowering blood sugar, which significantly improves insulin resistance (but liver and kidney function must be normal) [188]. TZDs mainly act through the nuclear receptor PPARγ, PPARγ is present in the main target tissues of insulin, such as liver, adipose tissue and muscle. Activation of PPARγ can regulate the transcription of insulin-responsive genes, thereby regulating glucose production, transportation and utilization [189]. TZDs mainly include rosiglitazone and pioglitazone. Rosiglitazone has been reported to increase the risk of cardiovascular diseases such as congestive heart failure and myocardial infarction [190]. Pioglitazone is another TZD that has not been reported to have such cardiovascular risk. Clinical trials have shown that over a 3-year period, there is a modest reduction in major cardiovascular events in high-risk diabetic patients when treated with pioglitazone. However, the safety of pioglitazone related to bladder cancer has also raised concerns, leading to safety warnings and drug withdrawals in parts of Europe [191].

### Glucagon-like peptide 1 (GLP-1) receptor agonists

Glucagon-like peptide -1 (GLP-1) is released from intestinal endocrine cells, which controls the dietary-related blood glucose deviation by increasing insulin release and inhibiting glucagon secretion [192]. However, endogenic GLP1 is easily degradable and has a short half-life. Therefore, researchers have developed GLP-1 receptor agonists compounds, which have the same function as GLP1, but can avoid degradation and thus function for a long time.

GLP-1 receptor agonists such as exenatide, liraglutide, exenatide stimulates insulin secretion in a glucose-dependent manner. In addition, GLP-1 receptor agonists can also play a hypoglycemic role by slowing down gastric emptying and suppressing appetite via central nervous system [193].

### Dipeptidyl peptidase 4 (DDP-4) inhibitors

Stimulated by nutrients, especially glucose, glucose concentration-dependent glucagon-like peptide-1 (GLP-1) is released from intestinal cells. After GLP-1 enters the blood, it can not only stimulate islet β cells to secrete insulin seretion but also inhibit the secretion of glucagon, resulting in lowering blood sugar level. Dipeptidyl peptidase-4 (DPP-4), a transmembrane glycoprotein possessing dipeptidyl peptidase activity, is to participate in the hydrolysis of GLP-1. Hydrolysis of GLP-1 can be inhibited by suppressing the activity of DDP-4 [194]. Sitagliptin is one of the inhibitors of DPP4 currently on the market, and has been approved in more than 130 countries around the world for the treatment of adult T2DM patients [194]. Extensive clinical evidence has confirmed the hypoglycemic efficacy of sitagliptin for T2DM patients with various complications. Moreover, sitagliptin is generally well tolerated, most adverse events are mild to moderate, and relatively few patients stop treatment because of these events [195].

### FGF21

Human FGF21 is mainly produced and secreted by the liver [196]. It mainly regulates monosaccharide intake and sweet food preference through FGF21 signaling [197]. The functions of FGF21 are mainly manifested in glucose metabolism, lipid metabolism and insulin resistance [198]. Pegbelfermin is a long-acting pegylated human recombinant FGF21 analog, which is currently in the phase 2 clinical research stage for the treatment of NASH and T2DM [199]. AKR-001 is another long-acting FGF21 analog developed by Akero Therapeutics, and it is currently in the phase 1 clinical trial stage for the treatment of T2DM [200].

### Agonists for G-protein-coupled receptors

G protein-coupled receptors include GPR40, GPR119 and GPR120, which are expressed in pancreatic β cells, and their endogenous ligands are long-chain and medium-chain free fatty acids [201]. The data from clinical trials of GPR40 agonist TAK-875 has proved that hemoglobin A1C (HbA1c) levels in T2DM patients can be reduced significantly after treatment with TAK-875. However, the development of TAK-875 has been terminated due to the hepatotoxicity among patients in phase 2 and 3 clinical trials [202]. Although there are many safety problems in developing small molecule GPR40 agonists for T2DM, drugs targeting this target are still under study.

## Conclusions and perspectives

Metabolic disorders cause damage to multiple organs and systems with complex pathologic alternations. Although animal studies and preclinical experiments have discovered plenty of novel targets and promising drug candidates for MASH, AH and diabetes, lots of new drug candidates are being examined alone in the clinical trials and the majority indicates invalid. Any single therapeutic agent may not be adequately potent to cure these diseases when considering the complicated pathophysiology [203]. Another possible reason for the failures of clinical trials is the animal models for these diseases at present don’t complete imitate clinical features of the patients. Although there are numerous genetically induced, diet-induced, and toxin-induced models of MASH, not all these models faithfully phenocopy and mirror the human pathology very well [204]. Patients with metabolic disorders have long courses and the long-term efficacy should be insured for MASH and diabetes, which will cause loss to follow-up. Currently, the gold standard for the assessment of MASH severity is liver biopsy, worsening the patient compliance during the clinical trials. These challenges should be taken into consideration when developing a new drug candidate.

Currently, the increasing burden of patients with MASH and ALD is threatening the health of people and the finances of countries. Due to the rarity of drug treatments for MASH and AH as well as the limited efficacy of the existing drugs, novel drugs and promising targets are promising. Potential treatments for MASLD should focus on long-term efficacy and safety, while new drugs for treating AH must focus on short-term outcomes. Given the complex pathophysiology of MASH syndrome and ALD, rational combination therapies targeting complementary mechanisms may provide a novel strategy for the comprehensive treatment of MASH syndrome and ALD. In addition, understanding the exact pathogenesis of patients with metabolic disorders may emphasize individualized treatment.

Although many drugs developed for T2DM have achieved initial results so far, it is still very difficult to cure diabetes, and the side effects greatly limit the use of the newly-developing drugs. With more and more targets discovered by researchers and deeper understanding of targets, more and more emerging technologies and drug candidates are being approved for clinical trials. As the mechanisms of T2DM is complicated and multifactorial, which tends to be diagnosed with MASLD, combined therapeutic strategies with newly drugs may provide a novel sight to this disease.

## Data Availability

Not applicable.

## Abbreviations

25HC3S: 25-Hydroxycholesterol 3-sulfate
AASLD: American Association for the Study of Liver Diseases
ADA: American Diabetes Association
AH: Alcoholic hepatitis
ALD: Alcohol-associated liver disease
ALT: Alanine aminotransferase
AMPK: 5ʹ-AMP-activated protein kinase
ASH: Alcoholic steatohepatitis
AST: Aspartate aminotransferase
CVC: Cenicriviroc
CVD: Cardiovascular disease
DAMP: Damage-associated molecular pattern
DM: Diabetes mellitus
DNL: De novo lipogenesis
EASL: European Association for the Study of the Liver
FDA: Food and Drug Administration
FGF: Fibroblast growth factor
FXR: Farnesoid X receptor
G-CSF: Granulocyte colony-stimulating factor
GLP-1: Glucagon-like peptide -1
HCC: Hepatocellular carcinoma
HDL-c: High-density lipoprotein cholesterol
HFF: Hepatic fat fraction
IL-1: Interleukin-1
KHK: Ketohexokinase
LDL-c: Low-density lipoprotein cholesterol
LPS: Lipopolysaccharide
MASH: Metabolic dysfunction-associated steatohepatitis
MASLD: Metabolic dysfunction-associated steatosis liver disease
NAFLD: Nonalcoholic fatty liver disease
NEFAs: Nonesterified fatty acids
OCA: Obeticholic acid
PAMP: Pathogen-associated molecular pattern
PNPLA3: Patatin like phospholipase domain-containing protein 3
PPAR-γ: Proliferator-Activated Receptor γ
SCD1: Stearoyl-CoA desaturase 1
SGLT: Sodium-glucose cotransporter
T1DM: Type 1 diabetes
T2DM: Type 2 diabetes
Th22: T helper 22
THR: Thyroid hormone receptor
TZDs: Thiazolidinediones
FABP4: Fatty acid-binding protein 4
FFA: Free fatty acid
FAS: Fatty acid synthase
SREBP-1c: Sterol regulatory element-binding protein-1c
SLC5: Solute carrier 5
CYP7A1: Cytochrome P450 7A1
NF-κB: Nuclear factor kappa-B
MUFAs: Monounsaturated fatty acids
HCD: High carbohydrate diet
HFD: High fat diet
TG: Triglyceride
WAT: White adipose tissue
*PPARG*: PPAR gamma
FGFR: FGF receptor
ERK1/2: Extracellular signal-regulated kinases 1 and 2
STAT3: Signal transducers and activators of transcription 3
BAT: Brown adipose tissue
*Ucp1*: Uncoupling protein 1
CREB: CAMP response element-binding protein
IL-22: Interleukin-22
DPP-4: Dipeptidyl peptidase-4
HbA1c: Hemoglobin A1C
PI3K: Phosphatidylinositol 3‑kinase
IRS: Insulin receptor substrate
GLUT4: Glucose transporter type 4
DAG: Diacylglycerol
PKC: Protein kinase C
